# Movement-related beta ERD and ERS abnormalities in neuropsychiatric disorders

**DOI:** 10.3389/fnins.2022.1045715

**Published:** 2022-11-23

**Authors:** Jaime Peter, Francesca Ferraioli, Dave Mathew, Shaina George, Cameron Chan, Tomisin Alalade, Sheilla A. Salcedo, Shannon Saed, Elisa Tatti, Angelo Quartarone, M. Felice Ghilardi

**Affiliations:** ^1^Department of Molecular, Cellular and Biomedical Sciences, CUNY School of Medicine, New York, NY, United States; ^2^IRCCS Centro Neurolesi Bonino Pulejo-Piemonte, Messina, Italy

**Keywords:** beta oscillations, EEG, MEG, movement, neurological diseases, psychiatric disorders, ERD, ERS

## Abstract

Movement-related oscillations in the beta range (from 13 to 30 Hz) have been observed over sensorimotor areas with power decrease (i.e., event-related desynchronization, ERD) during motor planning and execution followed by an increase (i.e., event-related synchronization, ERS) after the movement’s end. These phenomena occur during active, passive, imaged, and observed movements. Several electrophysiology studies have used beta ERD and ERS as functional indices of sensorimotor integrity, primarily in diseases affecting the motor system. Recent literature also highlights other characteristics of beta ERD and ERS, implying their role in processes not strictly related to motor function. Here we review studies about movement-related ERD and ERS in diseases characterized by motor dysfunction, including Parkinson’s disease, dystonia, stroke, amyotrophic lateral sclerosis, cerebral palsy, and multiple sclerosis. We also review changes of beta ERD and ERS reported in physiological aging, Alzheimer’s disease, and schizophrenia, three conditions without overt motor symptoms. The review of these works shows that ERD and ERS abnormalities are present across the spectrum of the examined pathologies as well as development and aging. They further suggest that cognition and movement are tightly related processes that may share common mechanisms regulated by beta modulation. Future studies with a multimodal approach are warranted to understand not only the specific topographical dynamics of movement-related beta modulation but also the general meaning of beta frequency changes occurring in relation to movement and cognitive processes at large. Such an approach will provide the foundation to devise and implement novel therapeutic approaches to neuropsychiatric disorders.

## Introduction

Almost a century after Berger’s first description of sensorimotor rhythms (Berger, 1929), a growing number of papers has established solid links between movement and specific dynamics of brain oscillations. Numerous is the evidence, obtained by non-invasive cortical recording techniques, such as electroencephalography (EEG) and magnetoencephalography (MEG), as well as by invasive cortical (electrocorticogram, ECoG) and subcortical recording techniques (from deep brain stimulation, DBS, devices). Changes in the electric or magnetic field over the sensorimotor cortex have been classified mostly based on their frequency. The first bandwidth described related to movement called *rolandic mu* rhythm or “central alpha” (8–12 Hz) (Magnus, 1954). Despite being characterized by similar frequency and amplitude as the occipital alpha oscillations, this rhythm was observed on centrally located scalp electrodes and its amplitude decreased (Gastaut, 1952), or desynchronized, over the sensorimotor area contralaterally to the moving effector for voluntary, imaged, passive, reflexive, and observed movements (McFarland et al., 2000). In the last 40 years, recordings with different electrophysiological techniques have shown that movements are also accompanied by changes over the sensorimotor areas in both beta (13–30 Hz) and gamma (30–200 Hz) bands (Pfurtscheller, 1981; Feige et al., 1996; Kilavik et al., 2013; Nowak et al., 2018). While gamma activity has a prokinetic nature since it increases during movement planning and execution (Muthukumaraswamy et al., 2010; Cheyne and Ferrari, 2013; Tatti et al., 2022), beta activity may have some “anti-kinetic” significance (Brown, 2006; Kilavik et al., 2013). Indeed, recordings over the sensorimotor areas show that beta power decreases or desynchronizes during the planning and the execution of movements, a phenomenon known as event-related desynchronization (ERD). Once movement is terminated, beta power starts increasing and reaches a peak with values greater than those at baseline, an occurrence known as event-related synchronization (ERS) (Pfurtscheller, 1981; Engel and Fries, 2010; Kilavik et al., 2013; Zaepffel et al., 2013; Spitzer and Haegens, 2017). These oscillations are present both over the sensorimotor cortex and in the basal ganglia, although controversies exist on whether they originate independently or are inter-dependent expression of the cortico-basal ganglia network activity (Spitzer and Haegens, 2017; Schmidt et al., 2019; Barone and Rossiter, 2021).

Despite extensive work in humans and animals, what is the functional role of the sensorimotor beta oscillations is a question still in need of an answer. The classic “idling” hypothesis views beta rhythm as an inhibitory frequency of the sensorimotor system (Pfurtscheller et al., 1996; Zaepffel et al., 2013). Accordingly, beta ERS and ERD would represent an “idle” state and an activation of motor cortex neurons, respectively. However, this hypothesis does not explain why similar modulation of beta power can also be observed during passive movements, motor imagery, and movement observation (Kilavik et al., 2013; Zaepffel et al., 2013; Tatti et al., 2019), as well as why its amplitude is not associated with specific movement parameters or effectors (Tatti et al., 2019), suggesting that beta modulation may occur in different domains, including the sensorimotor and cognitive domains (Engel and Fries, 2010).

Two new lines of evidence support this wider view of beta ERD/ERS, beyond their significance in motor planning and execution. First, during movements, the beta ERD/ERS dynamic has been observed not only over sensorimotor areas but also over frontal and pre-frontal areas (Moisello et al., 2015; Nelson et al., 2017; Ricci et al., 2019b; Tatti et al., 2021, 2020). In addition, that same pattern of beta ERD/ERS oscillation accompanies also working memory (Lundqvist et al., 2016; Miller et al., 2018) and focused attention tasks (Hanslmayr et al., 2014; Zavala et al., 2017), suggesting that beta ERD/ERS are not mere reflection of motor-related activity but their significance must extend to processes common to motor and cognitive functions. In this perspective, beta ERD would serve to release cortical inhibition, thus enabling movement execution or cognitive flow, while beta ERS would signal the need to maintain the current motor or cognitive set (Engel and Fries, 2010; Zaepffel et al., 2013; Spitzer and Haegens, 2017). Based on those observations, it has also been proposed that beta modulation might be a common denominator for attention-related processes, in both motor and cognitive domains, needed to filter out and cancel confounders or phenomena that are irrelevant to the event (Schmidt et al., 2019). Second, beta ERD-ERS could be interpreted from a metabolic perspective: a recent series of studies has shown that movement-related beta modulation, and in particular ERS amplitude, increases with practice with a cumulative effect that was more visible over the most involved brain areas and that returned to baseline after simple rest without sleep (Moisello et al., 2015; Nelson et al., 2021, 2017; Tatti et al., 2021, 2020). Therefore, ERS—and beta modulation in general—may represent an expression of energy consumption that is necessary for use-dependent and plasticity processes (Ghilardi et al., 2021). This view is supported by evidence in animals and humans that have been linking beta power changes to GABA and lactate changes (Jensen et al., 2005; Roopun et al., 2006; Yamawaki et al., 2008; Hall et al., 2011, 2010; Muthukumaraswamy et al., 2013; Rossiter et al., 2014b; Grønli et al., 2016) as well as to learning and plasticity (Hsu et al., 2011; Tan et al., 2016; Nelson et al., 2017).

No matter the precise significance of beta ERD, ERS, and movement-related beta modulation in general, all past studies have considered them as hallmark of the sensorimotor system functioning (Espenhahn et al., 2017) and thus, as possible biomarkers of motor symptoms in several neurological and psychiatric diseases. Indeed, movement-related beta activity has been extensively studied in patients with Parkinson’s disease (PD), the second most common neurodegenerative disorder that is still diagnosed with the appearance of motor symptoms, especially bradykinesia. Conversely, only a few studies focused on cognitive and behavioral disorders that are not specifically characterized by motor symptoms, such as Alzheimer’s disease (AD), schizophrenia, and physiological aging, conditions where subclinical motor or sensorimotor abnormalities can be experimentally detected. With this review, we wished to answer the following questions: Are abnormalities of movement-related beta ERD and ERS clearly and specifically linked to motor symptoms? Are these abnormalities present in pathological conditions other than classical motor disorders?

We thus focused on studies investigating movement-related ERD and ERS in the beta range during motor tasks performed with a limb and recorded with electrophysiological techniques with high time resolution (such as EEG, MEG, ECog, and DBS) that allows for differentiating phases of planning, execution and post-movement. The reviewed papers were on pathologies both specifically affecting the motor system [e.g., PD, dystonia, stroke, amyotrophic lateral sclerosis (ALS), cerebral palsy (CP), and multiple sclerosis, (MS)] and in conditions that are not strictly associated with motor disorders, including physiological aging, AD, schizophrenia, and obsessive-compulsive disorders (OCD). The research of the literature did not disclose studies about beta ERD or ERS in other psychiatric conditions, such as major depression.

The results of this review indicate that abnormalities of movement-related beta ERD and ERS have been described not only in classical motor disorders but also in pathologies that do not present with overt motor signs. Most importantly, such electrophysiological abnormalities do not appear to be specifically linked to motor symptoms but, in some instances, they were rather associated to non-motor problems. Altogether, the review of those studies suggests that beta ERD and ERS cannot be considered pure biomarkers of specific motor aspects, but they rather represent neural mechanisms common to features of motor and non-motor functions.

## Movement-related beta event-related desynchronization and event-related synchronization in neuropsychiatric diseases

Throughout the present paper, we use the terminology of beta ERD to indicate decreases of beta power before and during movement and beta ERS for increases of beta power after movements. A description of all the retrieved studies is reported in Tables 1–4. A summary of the results are in Table 5.

**TABLE 1:** Review of studies on movement-related beta ERD and ERS in stroke and ALS.

Authors	Rec.	Task	Subjects	Main Findings
**A. Stroke**				
Chalard et al., 2020	EEG	Elbow extension	15 subjects with different cortical or subcortical stroke sites. Testing: 8–116 months post stroke.	Reduced ERD correlated with increased activation of elbow flexors.
Shiner et al., 2015	MEG	Finger tapping	10 subjects with single stroke and upper limb motor deficit. Testing: 3–45 months post stroke.	Decreased ERD and ERS amplitude correlated with performance.
Rossiter et al., 2014a	MEG	Hand grip task	23 subjects with different stroke locations. Testing: 0.9–207.9 months post stroke.	Reduced ERD amplitude in lesioned M1 and lower ERD ratio in patients than controls. Greater impairment associated with reduced ERD amplitude in lesioned M1.
Espenhahn et al., 2020	EEG	Tracking and wrist movements	16 subjects with cortical or subcortical stroke. Testing: 41–220 months post stroke.	ERD amplitude 10% smaller in patients compared to controls.
**B. Amyotrophic lateral sclerosis (ALS)**				
Bizovièar et al., 2014	EEG	Sniffing test; right index flexion	21 subjects with ALS (2 bulbar onset, 19 spinal onset)	Reduced beta ERD amplitudes in ALS during both tests. Decreased beta ERS amplitude in ALS, ipsilaterally to the movement.
Bai et al., 2010	EEG	Finger extension; motor imagery task	3 subjects with ALS, 3 subjects with PLS, no normal control group	Presence of ERD and ERS in ALS and PLS. Decreased ERD and ERS in motor imagery task compared to finger movements.
Riva et al., 2012	EEG	Self-paced right thumb extensions	16 subjects with ALS (mean disease duration: 30 months)	No significant difference in ERD between groups. Reduced ERS in patients. ERS negatively correlated with corticospinal damage.
McMackin et al., 2021	EEG	Go/NoGo task with finger mouse click	24 ALS patients (median age: 69; range: 59–72 years); 33 age-matched normal controls	Reduced ERD during all trials in patients. Correlation between ECAS cognitive scores and ERS amplitudes.
Kasahara et al., 2012	EEG	Hand grasping motor imagery	8 subjects with ALS with various motor impairments	Reduced ERD in patients compared to controls. Worsening bulbar scales correlated with reduced ERD. ERS not reported.
Hosni et al., 2019	EEG	Motor Imagery task	6 subjects with ALS (mean age: 57.0 ± 15.7 years)	Reduced and delayed ERD in patients, particularly during right-hand MI. ERS not clearly detectable.

(A) Most studies found reduced ERD amplitude in post-stroke patients. (B) In general, ALS studies reported reduced ERD and ERS amplitude.

**TABLE 2:** Review of studies on movement-related beta ERD and ERS over cortical and subcortical structures in Parkinson’s disease with EEG, MEG, cortical (ECoG) and subcortical (STN DBS) recordings.

Authors	Rec.	Task	Subjects	Main Findings
Pfurtscheller et al., 1998	EEG	Self-paced movements	17 subjects with PD tested on-drug; mean age ± SD: 61.6 ± 6.1 years; PD duration: 3.8 ± 3.0	Reduced ERS in PD compared to controls.
Magnani et al., 1998	EEG	Self-paced movements	10 subjects with PD tested off-drug; PD duration: 2–9.2 years; age range: 44–77	Delayed ERD peak latency in PD compared to controls.
Brown and Marsden, 1999	EEG	Wrist movements	12 subjects with PD tested on- and off-drug; age range: 45–70	Drug treatment restores ERD to normal control group level; ERD improvement correlates with motor performance improvement.
Heinrichs-Graham et al., 2014b	MEG	Right index tapping	19 subjects with PD tested off-drug; disease duration: 1–9 years; age range: 52–77	Significantly diminished ERD in PD compared to controls.
Stegemöller et al., 2016	EEG	Index movements paced by 1–3 Hz tones	9 subjects with PD tested on- and off-drug; age range: from 53–77 years	Lower amplitude of ERD-ERS peak to peak amplitude in PD both off/on medications compared to controls.
Moisello et al., 2015	EEG	Arm reaching task	15 subjects with PD tested on-drug; mean PD duration ± SD: 6.7 ± 4.1 years; age: 60.7 ± 6.7	ERD-ERS peak to peak amplitude (beta modulation) reduced in PD. Attenuated ERS in PD. Lower practice-related beta modulation increase in PD compared to controls.
Nelson et al., 2017	EEG	Arm reaching task	11 subjects with PD tested on-drug; mean PD duration ± SD: 5.0 ± 2.1 years; age 59.1 ± 5.8	Decreased retention of motor skills and lower practice-related beta modulation increase in PD compared to controls; 24-h retention correlates with beta modulation increase.
Wu et al., 2019	MEG	Go/No Go task	12 subjects with PD tested off-drug; PD duration: 1–20 years; age range: 53–77	Reduced ERD in NoGo; reduced ERS during both Go and NoGo in PD. Delayed ERD and ERS during Go in PD compared to controls.
Vinding et al., 2019	MEG	Proprioceptive stimulation passive index movements	12 subjects with PD tested on- and off-drug; PD duration: 1–11 years; age range: 45–75	Reduced ERS in PD compared to controls. No change with medication
Labyt et al., 2005	EEG	Relaxation and contraction	16 subjects with PD; PD duration: 0.5–3 years; age range: 40–77	Delayed ERD peak and ERS disappearance during voluntary relaxation with the more affected limb.
Magnani et al., 2002	EEG	self-paced finger movement	14 subjects with PD tested on- and off-drug; PD duration: 2–6 years; age range: 54–78	Delayed ERD in akinetic PD patients compared to controls and improves after chronic drug treatment.
Praamstra and Pope, 2007	EEG	Choice response task	10 subjects with PD tested off-drug; PD duration: 6–9 years; age range: 56–68	Reduced ERD and ERS in PD compared to controls
Weersink et al., 2020	EEG	Instructed arm swing	17 subjects with PD	Reduced ERD during gait preparation and movement onset compared to controls.
McLinden et al., 2021	EEG	Self-paced finger tapping	8 subjects with PD tested on-drug; PD duration: 1–15 years; age range: 56–80	Reduced ERD in PD compared to controls.
Tamás et al., 2006	EEG	Self-paced on/off switch	10 subjects with PD on-drug, PD duration: 0.5–11 years, age: 41–80; 10 subjects with essential tremor, 0.5–20 years, 48–81; 10 normal subjects	No group differences. In PD, reduced ERS amplitude for the tremulus hand compared to the other hand. In essential tremor, delayed ERS for the most affect hand compared to the other one.
Johari and Behroozmand, 2021	EEG	Button press and speech tasks, predictable and unpredictable time	15 subjects with PD on-drug; Hoehn and Yahr: 1–3, median 1.5; age range: 60–75 years	PD were always slower than controls. Pre-movement beta ERD had similar time courses, but with between-task differences
Smith et al., 2012	EEG	Postural responses to backward surface translations	12 subjects with PD on-drug; mean PD duration: 77 years; mean age: 67.5	Greater ERD in PD compared to controls. Increased beta ERD in PD was associated with decreased adaptability of postural responses.
Labyt et al., 2003	EEG	Index extension and targeting motor task	12 subjects with PD tested off-drug; PD duration: 0.2–3 years; age range: 44–80	Reduced ERS in the more akinetic limb.
Devos et al., 2003a	EEG	Self-paced wrist flexions	10 subjects with PD tested on- and off-drug; median PD duration: 15 years; median age: 60	ERS amplitude decreases in advanced PD, but increases with Levodopa.
Devos et al., 2003b	EEG	Self-paced wrist flexions	10 subjects with PD tested on- off-drug and on- off-STN; PD duration: 7–23 years; age: 44–73	Both STN stimulation and medication increase ERD amplitude.
Rowland et al., 2015	ECog, STN-DBS	Reaching task	10 subjects with PD tested off-drug, PD duration: 4–15 years, age: 47–72; 8 subjects with essential tremor; disease duration: 6–50 years; age: 59–78.	Stronger ERD in PD compared to essential tremor in cortex.
Kondylis et al., 2016	EcoG	Visually cued hand grip task	11 subjects with PD off-drug, age range: 47–71 years; 6 subjects with epilepsy, 19–50 years; 9 subjects with essential tremor, 55–78 years	Greater ERD in PD compared to both control groups.
Canessa et al., 2016	EEG, STN-DBS	Arm reaching movements	7 subjects with PD tested off-drug; disease duration: 10–19 years; age range: 51–67	Stronger beta ERD and ERS in STN of the hemisphere with less striatal dopaminergic innervation during reaching.
Kühn et al., 2004	STN-DBS	Go/No Go task	8 subjects with PD tested on- and off-drug; PD duration: 10–22 years; age range: 50–71	ERD and ERS in the STN had pattern similar to that of cortical activity.
Kühn et al., 2006	STN-DBS	Wrist extension (ME), motor imagery and non-motor visual imagery tasks	8 subjects with PD tested off-drug; PD duration: 5–17 years; age range: 43–67	Movement and motor imagery produce in the STN similar ERDs that are larger than ERD elicited by visual imagery. ERS is larger during movement than during either motor or visual imagery.
Oswal et al., 2013	STN-DBS	Reward-complexity task: limb movements	9 subjects with PD tested on- and off-drug; PD duration: 8–19 years; age range: 26–62	Drug treatment increases ERD in the STN.
Doyle et al., 2005	STN-DBS	Joystick movements	14 subjects with PD tested on- and off-drug; PD duration: 8–18 years; age range: 43–73	Drug treatment increases ERD duration and magnitude. ERD latency and magnitude inversely correlate with degree of motor impairment.
Joundi et al., 2013	STN-DBS	Synchronized finger tapping	11 subjects with PD tested on-drug; PD duration: 2–17 years; age range: 42–70	ERD and ERS changes with the rate of finger tapping in the STN.

Most studies found reduced ERD and ERS amplitude compared to healthy controls. Studies with ECog and STN-DBS either had no control groups or comparison was made with patients with a different disease state.

**TABLE 3:** Review of studies on movement-related beta ERD and ERS in dystonia, multiple sclerosis, and cerebral palsy.

Authors	Rec.	Task	Subjects	Main Findings
**A. Dystonia**				
Crowell et al., 2012	ECoG	Movements of 5 different body parts	10 subjects with primary cervical or cranio-cervical dystonia, disease duration: 2–33 years; 11 PD subjects; PD duration: 6–20 years; 10 essential tremor subjects; disease duration: 15–42 years	Reduced ERD amplitude compared to PD and essential tremor patients.
Toro et al., 2000	EEG	Finger abduction	16 subjects with writer’s cramp	Reduced ERD amplitude in the 20–30 Hz frequency band.
van Wijk et al., 2017	MEG	Button Press	9 subjects with dystonia; disease duration: from 3 to 23 years. No controls.	Reduced ERD amplitude in lower beta frequency range.
Hess et al., 2020	EEG	Head turning task; Ballistic arm movement task; Pinch grip task	15 subjects with cervical dystonia (mean disease duration: 7.3 years); 23 subjects with dystonic tremor (6.4 years); 17 healthy age-matched controls	Reduced ERD amplitude in both cervical dystonia and dystonic tremor groups for both dystonic and non-dystonic muscles.
**B. Multiple sclerosis (MS)**				
Leocani et al., 2001a	EEG	Thumb extension	32 subjects with relapsing-remitting MS, no motor disturbances	Reduced ERS and ERD amplitude correlated with greater fatigue scores in MS.
Waldman et al., 2020	MEG	Button press	14 subjects with pediatric onset MS	ERS amplitude reduced in MS patients.
Arpin et al., 2017	MEG	Knee extension	15 subjects with relapsing-remitting or relapsing MS.	Reduced ERS amplitude correlated with task performance deterioration.
Barratt et al., 2017	MEG	Clicking task	18 subjects with relapsing-remitting MS and 3 with progressive MS	Increased ERS timing.
**C. Cerebral palsy (CP)**				
Kurz et al., 2020	MEG	Self-paced on/off switch	20 subjects with spastic diplegic presentation (mean age ± SD: 15.5 ± 3 years)	Increased ERD amplitude in CP; correlation with slower walking.
Kurz et al., 2017	MEG	Target force matching isometric knee extension	13 subjects with spastic diplegic presentation (mean age ± SD: 15.5 ± 3 years)	Increased ERD amplitude in CP in primary motor cortices, premotor area, inferior parietal lobe and inferior frontal gyrus. Reduced ERD in visual areas.
Kurz et al., 2014	MEG	Target force matching isometric knee extension	13 subjects with either spastic diplegia or hemiplegia (mean age: 14.25 years)	Increased ERD amplitude in CP.

(A) ERD amplitude is reduced in patients with dystonia. (B) Most studies found reduced ERS in MS, mostly in relation to fatigue. (C) In CP studies there is consensus about increased ERD amplitude.

**TABLE 4:** Review of studies on movement-related beta ERD and ERS in development and aging, Alzheimer’s disease, and schizophrenia.

Authors	Rec.	Task	Subjects	Main Findings
**A. Development and aging**				
Ricci et al., 2019a	EEG	Arm Reaching movements	13 younger adults (mean age ± SD: 24.2 ± 4.5 years); 13 older adults (57.5 ± 8.2 years)	Older subjects were slower and less accurate during movement. No difference is beta oscillatory activity between old and young group.
Johari and Behroozmand, 2020	EEG	Button pressing task	15 younger adults (age range, mean ± SD: 19–30; 22.6 ± 3.0 years); 15 older adults (50–77; 63.9 ± 8.6)	Greater ERD in older compared to younger adults. Faster reaction times correlated with weaker beta ERD.
Gaetz et al., 2010	MEG	Index finger movements	10 children (age range: 4.5–6.5 years); 10 pre-pubescents (11.5–13.5); 10 adults (24–42)	ERD and ERS amplitudes increases with age.
Gehringer et al., 2019	MEG	Isometric ankle plantarflexion target-matching task	22 adults (mean age ± SD: 36.6 ± 5.0 years); 21 adolescents (14.0 ± 2.1 years)	Reduced ERD and ERS after practice in adolescents, but was stronger in adults.
Rossiter et al., 2014b	MEG	Isometric hand grips	32 adults (mean age ± SD: 51 ± 21 years, range 22–82 years)	No difference between groups. However, age was positively correlated with ERD amplitude in ipsilateral M1.
Toledo et al., 2016	EEG	Button press in response to passive ankle dorsiflexion	19 young adults (age range: 21–32 years); 19 older adults (65–76 years)	ERD larger and delayed in older compared to younger adults. ERS found only in older adults. Earlier ERD and ERS associated with faster responses.
Mary et al., 2015	MEG	Auditory-cued key presses with 4 fingers, 5-element sequence	15 young adults (range 18–30 years; 24.3 ± 3.3); 14 older adults (range 65–75 years; 69.1 ± 1.5)	Enhanced ERS and ERD after learning in young compared to older adults.
Walker et al., 2020	MEG	Rapid leg dorsiflexion	11 young adults (mean age ± SD: 25 ± 3 years); 12 older adults (70 ± 3 years)	Stronger ERD post-proprioceptive stimulation in older compared to younger adults. No significant difference in ERS.
Sallard et al., 2016	EEG	Motor switching task; bimanual to unimanual finger tapping	17 young adults (mean age ± SD: 25 ± 3 years); 13 older adults (67 ± 4 years)	ERS increase in older compared to younger adults bilaterally over frontal and parietal areas.
Sailer et al., 2000	EEG	Finger extensions	8 young adults (age range, mean ± SD: 18–27; 24 ± 3.5 years); 8 older adults (55–78; 64.1 ± 9.6).	Increased activation of SMA in older in the high beta range.
Labyt et al., 2003	EEG	Finger extension and arm elevation	8 young adults (age range 25–35 years); 9 older subjects (55–65 years)	Decreased ERS amplitude in older subjects during targeting task.
Labyt et al., 2004	EEG	Self-paced finger tapping	8 young adults (age range: 20–35 years); 9 older adults (55–70 years)	Decreased ERS amplitude in older subjects compared to younger adults.
**B. Alzheimer’s disease (AD)**				
Babiloni et al., 2000	EEG	Finger extension	13 subjects with mild to moderate AD; diagnosis within 1–3 years. No motor deficits.	In AD, extra beta ERD activation of centromedial areas and extra beta ERS activation of ipsilateral rolandic area with frontal preponderance.
**C. Schizophrenia and obsessive-compulsive disorders**				
Hunt et al., 2019	MEG	Visuo-motor task with right index finger abduction	112 healthy subjects with schizotypal features	Schizotypal Personality Questionnaire score and ERS negatively correlated.
Gascoyne et al., 2021	MEG	Visuo-motor task: finger abduction	29 recent-onset (<5 years; no or minimal antipsychotics); 35 schizophrenic subjects (≥ 10 years; stable use of antipsychotics)	Reduced ERS in schizophrenia. ERS negatively correlated with severity of disease.
Robson et al., 2016	MEG	Self-paced button press	23 subjects with stable state schizophrenia	Reduced ERS in schizophrenia. No significant difference in ERD.
Leocani et al., 2001b	EEG	Self-paced right thumb movements	10 right-handed subjects with OCD (mean age: 28 years; disease duration: 6–28 years)	Reduced beta ERS amplitude in OCD. No significant difference in ERD amplitude or ERD/ERS latency.

(A) ERD is greater in adults than children; no clear effect of old age. (B) In AD, beta activity is spread. (C) Schizophrenia symptoms are linked to ERS decrease.

**TABLE 5:** Summary of the results of EEG and MEG studies in neuropsychiatric diseases.

	Studies (N)	ERD amplitude	ERS amplitude	ERD latency	ERS latency
Stroke	4	Reduced (4)	Reduced (1)		
ALS	6	Reduced (5)	Reduced (4)		
PD*	17	Reduced (9) Increased (1)	Reduced (8)	Delayed (5)	Delayed (1)
Dystonia	4	Reduced (4)			
MS	4	Reduced (1)	Reduced (3)		Delayed (1)
CP	3	Increased (3)	Reduced (3)		
Aging	10	Increased (5)	Decreased (2) Increased (2)	Delayed (2)	Delayed (2)
Development**	2	Increased (2)	Increased (2)		
AD	1	Extended topography (1)	Extended topography (1)		
Schizophrenia	3		Reduced (3)		
OCD	1		Reduced (1)		

Reported in this table are the number of studies (in parenthesis) that showed reduced and increased amplitudes or delayed latency ERD and ERS in the diseases compared to healthy controls with the number of studies supporting the finding in parenthesis.

*PD studies included in this table are only those that compared PD with healthy control groups with EEG and MEG recordings.

**Two articles studied ERD and ERS changes with healthy children (under the age of 18) compared to healthy adults.

### Stroke

Stroke is a leading cause of disability in the United States. Both ischemic and hemorrhagic stroke elicit widespread complications that depend on the degree of damage and the brain areas involved. Approximately 90% of post-stroke patients experience motor deficits that lead to disability and impediments in their daily living (Williams et al., 1999). Understanding the changes of beta oscillations and their functional relevance could thus help in tailoring effective rehabilitative strategies, including non-invasive brain stimulation techniques (Motolese et al., 2022). Here we discuss the findings of papers on movement-related beta ERD and ERS recorded with EEG or MEG in post-stroke patients during different types of motor tasks (Table 1A).

The majority of these studies focused on ERD, the biomarker that shows the most dramatic changes with stroke (Table 1A). Indeed, results show that stroke patients display reduced ERD in the ipsilesional hemisphere. In one MEG study, patients with various levels of impairment and lesion sites were tested from less than a month to 17 years after their first stroke (Rossiter et al., 2014a). In stroke patients, the amplitude of the ERD amplitude over the ipsilesional primary motor cortex was reduced and negatively correlated with the degree of motor impairment. In line with these results, an EEG study using a continuous tracking task and a simple wrist flexion-extension task found that the magnitude of ERD was on average 10% smaller in chronic stroke patients compared to controls (Espenhahn et al., 2020). Another MEG study in chronic stroke patients performing a finger tapping task confirmed the ERD amplitude reduction, while ERS was sometimes undetectable (Shiner et al., 2015). Greater ERD and ERS amplitudes were associated with better performance. In line with those findings, an EEG study focused on spastic co-contraction in post-stroke patients and reported a significant ERD decrease during elbow extensions. The ERD decrease correlated with an increased activation of elbow flexors that, in turn, reduced the active elbow motion. The authors concluded that these phenomena likely gave rise to the spastic co-contraction that is present in many stroke patients (Chalard et al., 2020).

In summary, the ERD reduction reported by these studies could be due to the inability to decrease beta power and thus to properly activate the motor cortex, thereby negatively impacting the descending motor signals and the resulting movement (Rossiter et al., 2014a). Adam et al. (2015) also suggested that stroke lesions may increase the variability of ERD onset, peak, and duration across trials, thus causing a reduced amplitude of the averaged ERD. Also, since increase of GABA-A in the primary motor cortex correlates with enhanced ERD, stroke lesions may affect GABA-A receptors or decrease the GABA concentration at the synaptic level (Adam et al., 2015).

Another important finding of these studies is that, following a stroke, the contralesional hemisphere seems to play a larger role during movement. In fact, a MEG study showed that the ERD amplitude ratio (ERD of lesioned M1/ERD of contralesional M1), an expression of interhemispheric asymmetry, was usually lower in patients compared to controls and negatively correlated with the degree of motor impairment (Rossiter et al., 2014a). A plausible explanation is that, in normal conditions, contralateral M1 activation also inhibits the opposite M1 in order to produce an appropriate movement. In case of stroke, the lesioned M1 cannot efficiently inhibit the contralesional hemisphere, thus explaining the increased ERD over the spared M1. On the other hand, the increased recruitment of the contralesional hemisphere shortly after stroke could function as a recovery or compensatory mechanism (Quandt et al., 2019).

In addition to amplitude, the temporal characteristics of movement-related beta ERD and ERS may also be of some significance to understand stroke-related changes and recovery mechanisms. Interestingly, Shiner et al. (2015) found that, in chronic stroke, the shorter the ERD duration the better was the degree of motor function. Conversely, ERS duration (which was not quantifiable in patients with greater motor impairment) was positively correlated with the degree of motor function (Shiner et al., 2015). While there is no clear explanation for these phenomena, one could speculate that the prolonged ERD is due to the loss of the appropriate neuronal population to be recruited, so that greater neuronal loss is associated with longer ERD duration and poorer motor performance.

In conclusion, the reviewed studies suggest that that abnormalities of ERD and ERS in stroke patients are somewhat related to abnormal motor function in general or specifically during the motor task.

### Amyotrophic lateral sclerosis

Amyotrophic lateral sclerosis is a progressive disorder characterized by the selective degeneration of both upper and lower motor neurons while the somatosensory system is spared by the degenerative process. The onset of clinical manifestation is insidious with focal weakness that progressively spreads to most muscles, eventually resulting in atrophy. Symptoms of ALS typically begin in the limbs, although about one third of cases involve bulbar muscles, heralded by difficulty chewing, swallowing, or speaking (Brown and Al-Chalabi, 2017). Because motor execution may not be plausible due to motor limitations, many studies resorted to motor imagery, a condition that still elicits ERD/ERS patterns albeit with reduced magnitudes in both normal controls (Pfurtscheller and Neuper, 1997) and in patients with ALS (Bai et al., 2010). The studies on ALS are reported in Table 1B.

Electroencephalography studies with either motor or imagery tasks have reported decreased ERD amplitude in patients with ALS (Bizovièar et al., 2014, McMackin et al., 2021). In particular, one study (Bizovièar et al., 2014) found that, despite normal task performance, patients with ALS displayed lower beta ERD and normal ERS amplitude compared to controls. However, unlike the controls, in ALS, ERS was absent over the hemisphere ipsilaterally to the moving finger. The authors explained this result as a consequence of degeneration of corpus callosum fibers (Van Zandijcke and Casselman, 1995; Filippini et al., 2010; Chapman et al., 2012). These findings differed from those of another study (Riva et al., 2012) reporting normal ERD in ALS and the presence of ERS asymmetry (Riva et al., 2012, see supplemental material) that was proportional to the degree of corticospinal damage. The contrasting results of the two studies could be due to differences in the selections of the range of beta band, the characteristics of ALS populations, and the tasks’ demands.

Decreased ERD amplitude in ALS have been also reported with motor imagery tasks (Kasahara et al., 2012; Hosni et al., 2019). One of those studies further showed a correlation of ERD amplitude with bulbar function—the worse the bulbar scale score, the smaller the ERD—but not with hand motor function (Kasahara et al., 2012). No clear ERS were detected in both imagery studies, probably due to the nature of the task (Kasahara et al., 2012; Hosni et al., 2019).

The reviewed studies indicate that, in general, the finding of abnormal ERD/ERS in ALS did not directly relate to motor dysfunction, the main problem of this disease. However, correlation between cognitive scores with ERS amplitude was reported at least in one study (McMackin et al., 2021). This suggests that movement-related beta ERD/ERS may reflect cognitive problems that often accompany ALS in the absence of motor task performance disruption.

### Parkinson’s disease

Despite mounting evidence that PD is preceded and accompanied by non-motor symptoms, its diagnosis is still made upon the appearance of motor signs. It is thus not surprising that movement-related beta oscillatory activity in PD has been the object of many investigations. Moreover, because of approved surgical and DBS therapeutical approaches, these investigations have also been conducted with invasive electrophysiological techniques. Briefly, most studies have revealed that PD is generally associated with increased beta power at rest throughout the basal ganglia-thalamo-cortical motor network (Moran et al., 2011; Oswal et al., 2013; Stein and Bar-Gad, 2013; Heinrichs-Graham et al., 2014a). With a few exceptions, most PD studies have also shown that treatments with levodopa and DBS restore beta power during the resting state to lower levels (Brown, 2006; Degardin et al., 2009; Stein and Bar-Gad, 2013; Vinding et al., 2019; Wu et al., 2019; Wang et al., 2020). Despite high levels of resting state beta power, the review of the papers reported in Table 2 indicates that the typical pattern of movement-related ERD-ERS beta modulation is usually maintained in PD. Nevertheless, many studies found abnormal movement-related beta activity over the sensorimotor cortices and also in subcortical structures (Heinrichs-Graham et al., 2014a; Moisello et al., 2015; Rowland et al., 2015; Canessa et al., 2016; Kondylis et al., 2016; Stegemöller et al., 2016; Nelson et al., 2017; Vinding et al., 2019; Wu et al., 2019). In Table 2 we summarize the results of studies about cortical and subcortical movement-related beta ERD and ERS in PD with invasive and non-invasive recordings.

With the introduction of recording electrodes in the subthalamic nucleus (STN) or globus pallidum (Gpi) in the context of DBS treatment, many studies have focused on defining the pattern of electrical activity that are characteristic of PD in these nuclei (Kühn et al., 2004; Brown, 2006; Weinberger et al., 2009; Giannicola et al., 2010; Jenkinson and Brown, 2011; Moran et al., 2011; Stein and Bar-Gad, 2013; Heinrichs-Graham et al., 2014a; Wang et al., 2014; Canessa et al., 2016). Studies have revealed prominent oscillations in the beta frequency range (11–30 Hz) at rest (Brown, 2006; Weinberger et al., 2009). The pattern of movement-related beta oscillations in the STN has been clearly recorded with Go/NoGo tasks and motor imagery and it closely resembles that recorded from cortical sites (Kühn et al., 2004, 2006). Dopaminergic medication and STN DBS modulate beta oscillatory activity in PD patients, ameliorating motor symptoms (Devos et al., 2003a,b; Doyle et al., 2005; Oswal et al., 2013). Indeed, following levodopa administration, ERD amplitude increases in the STN with faster response times, regardless of task complexity (Oswal et al., 2013), while ERD latency decreases (Doyle et al., 2005). Clearly, definite conclusions about the effect of the disease cannot be directly drawn from ECoG studies because group comparisons can be performed in subjects with pathological basal ganglia activity and not with normal subjects. The small number of patients (from seven to eleven in most of the studies) and the great variability of the clinical phenotype are additional limitations of these studies. Nonetheless, studies with ECoG and STN DBS are useful to determine accurate topography, to find proper and personalized tuning stimulation parameters and, at the same time, to measure the behavioral output. Therefore, in this context, they could provide valuable information to guide and implement novel therapeutic interventions, such as adaptive stimulation (see: Pozzi and Isaias, 2022).

Besides the results from DBS studies, most of the EEG, MEG, and ECoG studies have found ERD abnormalities in PD. One of the reported alterations is a delay in ERD occurrence (Magnani et al., 1998; Labyt et al., 2005; Wu et al., 2019) that can be corrected with levodopa administration (Magnani et al., 2002). Many studies reported reduced beta ERD amplitude over the sensorimotor cortices of PD patients compared to different control groups with EEG, MEG, and ECoG recordings (Praamstra and Pope, 2007; Heinrichs-Graham et al., 2014b; Moisello et al., 2015; Canessa et al., 2016; Nelson et al., 2017; Vinding et al., 2019; Wu et al., 2019; Weersink et al., 2020; McLinden et al., 2021; Weersink, 2021). However, a few studies have either found no difference between patients with PD and patients with essential tremor (Tamás et al., 2006) or larger ERD amplitude in PD compared to essential tremor, epilepsy (Kondylis et al., 2016), and normal controls (Smith et al., 2012; Kondylis et al., 2016; Stegemöller et al., 2016; Johari and Behroozmand, 2021). Briefly, many factors could have played a role in the diverse results, such as differences in the study design, the analytical approach, the choice of the control groups, the small sample of subjects, the great number of tested variables as well as large within- and between-group variabilities in terms of age, performance, and number of recordings.

Only a few PD studies focused on movement-related beta ERS with EEG or MEG recordings. The general finding is that ERS amplitude is decreased in PD patients. This has been observed with active limb movements during an advanced cued task (Heinrichs-Graham et al., 2014b), a reaction time task (Moisello et al., 2015; Nelson et al., 2017), a Go/No-Go task (Wu et al., 2019), as well as with passive movements (Vinding et al., 2019). Reductions of beta ERS amplitude are already visible in the early stages of PD (Pfurtscheller et al., 1998). Studies on the performance of the two upper limbs showed that ERS amplitude is lower over the hemisphere contralaterally to the more akinetic limb (Labyt et al., 2005, 2003). Like for ERD, both dopaminergic therapy and DBS can acutely increase beta ERS amplitude; such increases usually correlate with the degree of motor improvement, especially for bradykinesia (Canessa et al., 2016). However, a study with passive movements reported administration of levodopa improved motor symptoms but it did not change ERS amplitude (Vinding et al., 2019).

A few studies have recently investigated the effect of motor practice on movement-related beta ERS in patients with PD and in normal controls. The authors found that beta ERS amplitude and movement-related beta modulation (defined as the difference from peak ERD to peak ERS) increase with practice over frontal and sensorimotor regions (Moisello et al., 2015; Nelson et al., 2017; Marchesi et al., 2019; Ricci et al., 2019a,b; Tatti et al., 2021, 2020, 2019). Moreover, the increase of beta ERS and modulation depth achieved at the end of practice predicted motor improvement measured 24 h later (Nelson et al., 2017), thus suggesting that practice-related ERS increases reflect some aspects of plasticity. In line with this conclusion, in PD, which is usually associated with reduced plasticity and retention (Marinelli et al., 2017), the practice-related ERS increase is significantly less evident, despite normal values of ERS and beta modulation at baseline (Moisello et al., 2015; Nelson et al., 2017). In summary, these works support the hypothesis that practice-related increases of beta ERS may represent local processes needed to engage plasticity-related activity, as recently hypothesized (Ghilardi et al., 2021).

### Dystonia

Dystonia is characterized by involuntary muscle contractions that may cause abnormal posture or repetitive movements with highly variable clinical features. The distribution and number of affected body parts range from a single location (focal dystonia), to two or more adjacent locations (segmental dystonia) or to all the areas of the body (general dystonia). While some motor manifestations of dystonia appear in isolation, others arise in association with other diseases or non-motor symptoms (Quartarone and Ghilardi, 2022). In Table 3A we report the summary of studies on movement-related beta oscillatory activity.

In general, patients with dystonia present with a broad reduction of beta ERD documented with different recording modalities. ERD amplitude reduction between 20 and 30 Hz was first reported in patients with writer’s cramp performing a finger abduction task during EEG recordings (Toro et al., 2000). Other studies have confirmed this result with many techniques. For instance, an EEG study investigated two cohorts of dystonic patients—one with cervical dystonia and the other one with dystonic tremor—while they performed a motor task; results showed that the amplitude of beta ERD was significantly reduced compared to healthy controls. An earlier ECoG study showed a similar reduction of ERD amplitude in patients with dystonia compared to patients with either PD or essential tremor (Crowell et al., 2012). In both studies, ERD decrease was present also for movements with non-affected muscles, suggesting that dystonia is associated with a generalized sensorimotor impairment in agreement with the results of kinematic and TMS studies (Pelosin et al., 2009; Quartarone and Hallett, 2013). Lower beta ERD amplitude in idiopathic dystonia was also confirmed by a study of local field potentials from GPi DBS with simultaneous MEG recording during a finger task (van Wijk et al., 2017). Greater coherence between DBS and MEG recordings in beta frequency range was associated with increased reaction time, confirming the role of beta oscillations in motor planning.

### Multiple sclerosis

Multiple sclerosis is an inflammatory neurodegenerative disease characterized by demyelination and widespread damage to the central nervous system. Therefore, the symptoms associated with this disease are diverse and often include motor and sensory impairments, visual disturbances, and fatigue with a highly variable time course. Magnetic resonance imaging (MRI) is the gold standard for diagnosing MS and for monitoring structural changes overtime, although the degree of demyelination cannot always predict disease progression (Daumer et al., 2009; Hemond and Bakshi, 2018). Electrophysiological measures have been used to supply further information about functional changes over time and to assess the effects of treatments. In Table 3B we report MS studies on movement-related beta modulation.

In general, MS is not associated with ERD alterations, while abnormalities in ERS have been usually reported by both EEG and MEG studies. Indeed, two MEG studies with different motor tasks found no difference in ERD amplitude between healthy controls and patients with different forms of MS (Arpin et al., 2017; Waldman et al., 2020). These results are in agreement with those of an earlier EEG study investigating a group of normal subjects and two groups of patients with MS, one with fatigue and the other without fatigue assessed with the Fatigue Severity Scale (Leocani et al., 2001a). While ERD amplitude was similar in non-fatigued MS patients and healthy controls, the fatigued MS patients displayed a smaller ERD than the other two groups. The greater fatigue scores corresponded to a larger ERD reduction, suggesting that non-motor features may contribute to ERD amplitude.

All the three studies also reported a decrease of ERS amplitude in MS. In one of them (Arpin et al., 2017), weaker ERS in MS patients correlated with abnormal task performance parameters. The study about the effect of fatigue (Leocani et al., 2001a), besides decreased ERS in MS compared to normal controls, found the smallest ERS amplitude in the fatigued MS group and a negative correlation between fatigue scores and ERS amplitude. Finally, a MEG report (Barratt et al., 2017) studying the temporal profile of beta power dynamics during a visuomotor task showed increased ERS timing in MS patients, confirming a disruption of beta ERS mechanisms. The authors further suggested that neuronal damage in MS, either from decreased neuronal density or demyelination, may lead to a decrease in neuronal firing and thus to a reduced ability to synchronize.

### Cerebral palsy

Cerebral palsy is the most prevalent cause of neurological impairment in the United States pediatric population, resulting from injury or improper development of part of the brain and presenting with both motor and sensory abnormalities. Periventricular white matter damage in CP during or shortly after birth may reduce the efficacy of information transmission along the thalamocortical and corticospinal tracts resulting in a wide variety of sensorimotor impairments and abnormalities in movement-related beta oscillations (Kurz et al., 2017).

A review of papers on movement-related beta modulation in CP (see Table 3C) shows that CP is in general associated with increased beta ERD. The MEG studies by Kurz and colleagues with spastic diplegic and hemiplegic CP patients during a goal-directed knee extension task (Kurz et al., 2014, 2017, 2020) all reported greater ERD amplitude in subjects with CP compared to age-matched controls. When auditory tones paced the movements, the ERD increase was widely spread, from the medial post-central gyrus to the superior parietal lobe (Kurz et al., 2014). ERD increases were also present in the primary motor cortices, premotor area, inferior parietal lobe, and inferior frontal gyrus when a visual feedback of their performance was given (Kurz et al., 2017). The authors concluded that the higher ERD amplitude within the M1 may indicate difficulty in monitoring the consequences of the ongoing motor actions and in matching their actions to the targets. The same study also showed decreased ERD amplitude over the occipital and other visual areas that was associated with slower reaction times and target matching error, thus suggesting that such reduction may reflect greater difficulty in performing visuomotor tasks. A similar MEG study with a task that did not provide online visual feedback but only knowledge of the results, confirmed the presence of ERD with greater amplitudes in the sensorimotor areas in CP compared to controls but no differences in ERD amplitudes over the occipital areas (Kurz et al., 2020). The authors stated that the lack of difference in terms of occipital beta ERD is possibly related to intact occipital function in this group of participants compared to the previous one. An alternative explanation should take into account the lack of online visual feedback during the task compared to the other studies. Finally, this study found that, in CP, greater ERD amplitude was associated with slower walking cadence, a relationship not found in controls, suggesting that increased ERD amplitude in CP may result from abnormalities in energy regulation.

### Normal aging

While several works have investigated the effect of aging with different types of motor tasks, only a few studies have addressed the evolution of movement-related beta oscillations during development (Table 4A). In particular, a MEG study by Gaetz et al. (2010) demonstrated that the amplitudes of beta ERD and ERS change as a function of age: both beta ERD and ERS are weaker in children (aged from 4 to 6 years) compared to adolescents (from 11 to 13 years), which, in turn, display weaker values than adults (from 24 to 42 years). The authors concluded that weaker beta ERD and ERS in children may reflect reduced motor cortical inhibition and thus high plasticity, which are typical of childhood and may serve to promote normal development. These findings are in agreement with the results of a more recent MEG study investigating differences between adolescents (mean age: 14.0 years) and adults (36.6 years) after practice sessions in a leg force task (Gehringer et al., 2019). At baseline, adolescents exhibited weaker beta ERD and ERS than the adult group over the contralateral sensorimotor cortex; after practice, beta ERD and ERS increased mostly in the adults (Gehringer et al., 2019).

With aging, and mostly in the later part of adult life, beside cognitive decline, motor abilities usually deteriorate, as shown in a variety of experimental tasks (Salthouse, 1984; Ranganathan et al., 2001). There are also reports indicating that, with aging, loss of motor cortical and spinal neurons can occur (Doherty et al., 1993; Eisen et al., 1996) together with sarcopenia (Priyadarsini et al., 2022). Aging-related performance deterioration can be accompanied by changes in movement-related beta oscillations (Mary et al., 2015; Sallard et al., 2016; Toledo et al., 2016; Walker et al., 2020), although a few studies failed to find any age effects (Labyt et al., 2003; Rossiter et al., 2014b; Ricci et al., 2019a; Table 4A). Briefly, reports of increased beta ERD amplitude in older compared to younger subjects have been described by both EEG and MEG studies in a variety of motor tasks over areas contralateral to the involved effector: during finger tapping (Sailer et al., 2000; Mary et al., 2015), button press (Johari and Behroozmand, 2020), speech (Johari and Behroozmand, 2020), ankle movements (Toledo et al., 2016), and involuntary stretch of leg muscles (Walker et al., 2020). Many of these papers also reported delayed timing of ERD.

Controversial results have been found for beta ERS. Early studies by Labyt et al. (2004, 2003) reported decreased ERS after self-paced finger movements in older compared to younger subjects. On the other hand, during assessment of ankle proprioception, the presence of ERS was noted only in the older group, but not in the younger group (Toledo et al., 2016). The authors interpreted this finding as a mechanism to overcome age-related deterioration of peripheral and central structures to generate an adequate sensorimotor output. In line with these findings, Sallard and coworkers (Sallard et al., 2016) found that older populations exhibited stronger beta ERS than younger populations over frontal and sensorimotor regions during finger tapping tasks. In contrast with these results, a study investigating age-related differences after proprioceptive stimulation showed increased ERD in the older group, but no group differences in terms of beta ERS (Walker et al., 2020). Despite differences in ERS timings, movement accuracy, and speed, reaching movements studies did not find age-related differences for both ERD and ERS amplitude (Ricci et al., 2019a). In addition, the study showed that the amplitude of ERS increased with practice in both groups at the same rate (Ricci et al., 2019a).

The contrasting results of these papers could be due to differences in tasks, sample size, and age ranges. Most importantly, age may not be the best predictor of cortical changes since other individual factors play important roles in aging processes, such as motor practice, lifestyle, gender, genetics, and differences in plasticity and energy mechanisms (Dachtler and Fox, 2017; Ricci et al., 2019a).

### Alzheimer’s disease

Alzheimer’s disease is one of the most common neurodegenerative disorders, characterized by a progressive decline in memory and cognitive function. Usually, motor dysfunctions are not clinically evident in either the early stages of AD or Mild Cognitive Impairment. Clinically evident motor impairment usually occurs in later stages of AD and manifests as apraxia, the inability to conceptualize and perform meaningful actions. Nevertheless, research studies have shown that subtle signs of motor impairment, such as increased reaction time (Koller et al., 1984; Müller et al., 1991), slow movements, impaired walking and balance (Buchner and Larson, 1987; Alexander et al., 1995; Ott et al., 1995) are already present early on in the disease. In particular, there is evidence that, in the early phases of AD, reaching movements are slower with a great reliance on continuous on-line visual cues and a scarce dependence on motor planning (Bellgrove et al., 1997; Ghilardi et al., 2000, 1999). Indeed, while accuracy of visually guided movements is similar in patients with AD and controls, movement speed and transport phase are significantly reduced in AD. When visual feedback is withheld, patients with AD also show a decay of movement accuracy. The degrees of motor performance disruption and cognitive abnormalities are highly correlated, thus pointing to impairments in frontal and parietal regions, a pathological hallmark of AD (Ghilardi et al., 2000, 1999). Therefore, early motor abnormalities may represent initial, subclinical manifestations of apraxia in AD. Despite the evidence of early impairment of motor function, there is only one EEG study in AD (Table 4B) directly investigating its neural bases and its possible association with movement-related beta oscillations (Babiloni et al., 2000).

In that study, EEG was recorded in patients with mild to moderate AD while performing movement extensions with the right middle finger (Babiloni et al., 2000). The results were compared to a group of age-matched healthy controls and a younger group. In agreement with previous reports, the movements of patients were slower than those of both control groups. Nevertheless, beta ERD and ERS amplitudes in the centroparietal electrodes contralaterally to the movement were similar in all three groups. Further analyses with surface Laplacian estimates of ERD and ERS showed a more extended activation in the AD group; differently from the control groups, ERD and ERS were spread to frontal areas and contralateral sites. No correlation was found between motor performance indices and ERD/ERS abnormalities. The authors concluded that to perform simple finger movements, AD patients may engage additional areas outside the classical motor cortical network, a pattern that is normally used for more complex movements. That supplementary recruitment is probably due to depleted resources in the sensorimotor network, suggesting that AD is “a global brain network disease” that includes abnormal “processing of sensorimotor information despite no overt movement disorder” (Babiloni et al., 2000).

### Schizophrenia and obsessive-compulsive disorders

Schizophrenia is defined by a range of symptoms that include distortion of reality, disorganized thought and behavior, and imbalance of dopaminergic pathways. Beside disorganization and psychomotor motor poverty, subtle signs of motor and sensory abnormalities can be present early on in the disease in a variety of motor activities, including gaze (Calkins et al., 2008), force adjustment and fine motor function (Rosen et al., 1991; Exner et al., 2006; Teremetz et al., 2014; Walther and Mittal, 2016; Térémetz et al., 2017), as well as gait and posture (Kent et al., 2012; Bernard et al., 2014). While antipsychotic medication could play a role (Putzhammer et al., 2005; Nowak et al., 2013), sensorimotor impairments can be seen in unmedicated patients and just after the first episode of psychosis (Caligiuri and Lohr, 1994; Wolff and O’Driscoll, 1999; Ayehu et al., 2014; Teremetz et al., 2014). On these bases, to explore the neural bases of schizophrenia-related sensorimotor abnormalities, a few studies investigated movement-related beta ERD and ERS in patients with schizophrenia and in normal subjects with different scores of schizotypal personality (Table 4C).

There are only two works investigating movement-related beta oscillations in patients with clinically diagnosed schizophrenia. Both studies were conducted with MEG and showed similar beta ERD amplitudes in patients and controls. Importantly, they both also reported a reduction of beta ERS amplitude that correlated with disease severity. In the first study, patients with stable schizophrenia and without motor problems performed self-paced button clicking movements after target presentation (Robson et al., 2016). The other study included a group with recently developed schizophrenia and absent or minimal antipsychotic medications and another with established psychosis (Gascoyne et al., 2021). They performed a finger abduction task in response to a stimulus. Both studies found decreased ERS which was associated with disease progression, with no significant role of medication. The evidence that the prime driver of beta ERS reduction is not medication but rather disease progression is also suggested by the results of another more recent study on schizotypal personality (Hunt et al., 2019). Schizotypy has been recognized as a set of traits quantitatively within normal range, but qualitatively similar to those of neurodevelopmental and schizophrenia spectrum disorders. In a sample of more than 100 normal subjects, the authors found that the degree of schizotypal personality was related to ERS amplitude decreases, in that lower ERS amplitude corresponded to higher Schizotypal Personality Questionnaire scores, with higher variance accounted for by the scores of disorganization and interpersonal factors (Hunt et al., 2019).

Obsessive-compulsive disorders is an anxiety disorder characterized by inability to suppress intrusive thoughts and repetitive actions with dysfunction of orbitofrontal cortex, anterior cingulate, basal ganglia, and other limbic structures. One study has shown decreased amplitude of beta ERS in a small cohort of subjects with OCD, a finding that was interpreted as impairment of post-movement deactivation mechanisms likely due to orbito-frontal cortex dysfunction (Leocani et al., 2001b; Table 4C).

## Discussion

This review suggests that neurological and psychiatric disorders with or without overt motor problems present with abnormalities of movement-related beta ERD and ERS and that such abnormalities are, in many cases, not directly related to motor symptoms. As summarized in Table 5, most studies found decreased ERD amplitude in stroke, ALS, dystonia, and PD; decreased ERD and ERS amplitudes were described in MS, especially in patients with fatigue; conversely, ERD with abnormally increased amplitude was reported in CP. As far as physiological maturation, a process accompanied by changes in motor and cognitive function, ERD and ERS amplitudes increase during development, reaching a plateau in adulthood, a finding that has been linked to the reduced cortical inhibition and high plasticity typical of childhood. However, contrasting results about the effects of old age suggest that age *per se* is not associated with changes in movement-related beta oscillations and other factors related to lifestyle, history, cognition, and perhaps genetics may play significant roles. Only a few studies have investigated possible changes in neuropsychiatric conditions without clinically evident sensorimotor impairment. In AD, ERD, and ERS associated with simple finger movements were spread beyond the normal motor cortical network over areas that, in normal subjects, are engaged only for complex movements. Such an enlarged recruitment suggests that first, neurodegeneration in AD must also affect the sensorimotor function, at least in terms of cortical re-wiring, and, second, that overt apraxia, which is often present later on in the disease, may stem from these early changes. Decreased ERS amplitude correlated with the progression of schizophrenia and also with the degree of schizotypal personality; in OCD, similar ERS findings were not related to motor performance. In summary, abnormalities of movement-related beta ERD and ERS were found in all the neuropsychiatric diseases we have reviewed and such abnormalities were not necessarily dependent on the presence of motor symptoms.

### Limitations and caveats

A few factors limit the interpretation of the findings of the reviewed studies. Some effects could have been underestimated because in many studies a small number of subjects were tested, while in others there was high variability of either the lesions’ location (as in the case of stroke) or the clinical characteristics of the disease (as in PD). Limitations include: different definitions of the beta band (for instance, in some studies from 13 to 25 Hz, in others from 15 to 35 Hz); *ad hoc* analyses focused on selected sub-bands (e.g., Johari and Behroozmand, 2021); single reports that require further confirmation, as in the case of AD, CP, and OCD. Another caveat is the interpretation of LFP recordings from cortical and subcortical sites in terms of ERD and ERS as reflecting the modulation of the strength of a sustained oscillation. Indeed, evidence suggests that beta ERD and ERS may likely represent the probability of occurrence of a brief bursting transient event (Canolty et al., 2012; Rule et al., 2017). Therefore, the common and current approach of averaging trials may not provide a thorough picture of beta activity and thus limits the understanding of beta ERS and ERD in the sensorimotor system. Also, ECoG and subcortical recording studies can provide information on a restricted topography and with comparisons of patients affected by other brain pathologies, thus limiting conclusions about the effects of the pathology itself. An example is the comparison of dystonia and PD recordings in the STN. The two diseases share many pathophysiologic features, such as alterations in movement-related beta oscillations, although STN DBS recordings showed lower beta ERD in dystonia compared to PD (Crowell et al., 2012). Nevertheless, these studies are useful to assess the effects of pharmacological or other therapy when associated to the clinical evaluation of the patient. Finally, we could not retrieve any study regarding beta modulation in other psychiatric conditions. These pathologies warrant further research to provide more information about the link between beta modulation and behavioral symptoms at large. This review did not take into account clinical studies related to traumatic brain injuries (TBI). To our knowledge, there are no systematic studies on TBI. Nevertheless, ERD/ERS could provide an effective mean for follow up studies in larger cohorts of patients with TBI or other diseases, as suggested by a single case study with a patient with open TBI (D’Arcy et al., 2020).

### Beta modulation in the sensorimotor cortex and beyond

While investigations in neuropsychiatric diseases have demonstrated impairments in movement-related beta oscillation, beta ERD and ERS cannot be considered markers of a specific status, disease, sensorimotor symptom, or brain lesion site. Indeed, the amplitude of ERD and ERS in normal subjects is not linked to specific kinematic characteristics (Tatti et al., 2019), a link that is instead evident for gamma power (Tatti et al., 2022), but it is probably related to other factors including practice and learning (Schmidt et al., 2019; Ghilardi et al., 2021). Therefore, it is likely that beta ERD and ERS have a wider meaning and reflect general abilities or mechanisms within the sensorimotor system –and associated areas at large- to ideate, assemble, program, promote and finalize a behavior as well as to update internal models related to these processes. Some findings reported in this review support this wider view. First, beta ERD and ERS can be elicited over the sensorimotor areas not only during the execution of movements but also during motor imagery without producing a measurable motor output, as confirmed by some ALS studies. Second, the ERD and ERS abnormalities found in AD (which did not correlate with any motor performance indices) may represent early phases of apraxia, a symptom likely resulting from progressive impairment of the objective Euclidean and body-centered spaces followed by the disruption of the actual space of object manipulation. Third, decreased beta ERD and ERS amplitudes were highly correlated with fatigue in MS, but not with motor disability. Indeed, fatigue is a symptom that cannot be confined to the motor domain but extends to mood and sleep regulation, as well as to other functions. Also, studies link fatigue to frontal areas dysfunction (Morgante et al., 2011). Fourth, movement-related beta ERS amplitude recorded over the sensorimotor cortex seems to be related to the degree of schizotypy and, in particular, to organizational and interpersonal factors (defined in the Schizotypal Personality Questionnaire) that heavily rely on frontal lobe function (Hunt et al., 2019). Finally, ERS abnormalities in ALS correlated with cognitive and behavioral scores and not with motor performance (McMackin et al., 2021). Other lines of evidence also support the notion that the beta ERD-ERS dynamics may have meanings that transcend motor function *per se*, linking it to attention-related and other processes. Indeed, movement-related beta modulation has been observed also over frontal and pre-frontal areas (Moisello et al., 2015; Ricci et al., 2019b; Tatti et al., 2019, 2020, 2021), suggesting a more diffuse involvement of cortical areas in movement programming, execution, stopping, and possibly in internal model updating. Also, ERD-ERS patterns in the beta range has been recorded in prefrontal areas even in non-motor tasks, specifically during both working memory (Lundqvist et al., 2016; Miller et al., 2018) and focused attention tasks (Hanslmayr et al., 2014; Zavala et al., 2017). For these reasons, as suggested by Schmidt et al. (2019), it is likely that cognition and movement share common processes –both regulated by beta modulation – that allow for focusing and suppressing irrelevant phenomena, with a sort of center-surround suppression mechanism of action. This view of common mechanisms underlying motor and cognitive processes, such as those expressed by beta modulation, transcends the conceptual separation of the different brain functions into motor and cognitive categories and should ultimately guide new research approaches for finding more effective therapeutical solutions.

### Movement-related beta modulation and practice

Finally, an aspect that only a few studies highlighted is that in normal subjects, beta ERS and ERD amplitudes increase with practice across trials (Moisello et al., 2015; Nelson et al., 2017), possibly reflecting plasticity-related phenomena. This interpretation is in agreement with the results of MEG studies (Mary et al., 2015; Gehringer et al., 2019) showing enhanced beta ERS after intensive motor training and with the finding that iTBS, a protocol inducing LTP-like phenomena in the sensorimotor system, enhances movement-related beta ERS in a subsequent motor task (Hsu et al., 2011). Furthermore, the increase is local and task-related, as beta ERS is enhanced over both frontal and somatosensory areas following visuo-motor learning, but not after practice in a purely visual learning task (Tatti et al., 2020) or in a simpler motor task without visuo-motor learning (Tatti et al., 2021). Such practice-related beta increase vanishes after a similar period (about 90 min) of either quiet wake or napping (Tatti et al., 2020), suggesting that movement-related beta modulation may reflect phenomena needed to start, maintain, and finalize LTP processes (Ghilardi et al., 2021). These conclusions about a link between beta power with learning and plasticity are also in agreement with the work by Tan et al. (2016). Intriguingly, as animal studies have shown a relation between beta power during movement and lactate consumption (Grønli et al., 2016), it is possible that practice-related beta changes may represent an index of local energy consumption and availability (Ghilardi et al., 2021).

### Future perspectives

Based upon this review and considerations derived from it, it is evident the need of further integrated research on beta ERD and ERS before these measures can be effectively used as biomarkers of specific brain functions in neuropsychiatric diseases. These future studies will be fundamental to determine: ERD/ERS general mechanisms and significance, the behavioral counterparts, the pharmacology, the topography and its dynamics, the biochemical, metabolic and electrophysiological processes involved at the subcellular, cellular, and system levels. For instance, from a pharmacological point of view, the amplitude of beta power and ERD have been linked to levels of GABA (Gaetz et al., 2010; Hall et al., 2011; Muthukumaraswamy et al., 2013) suggesting a relationship between beta oscillations and the balance between inhibitory and excitatory processes, and the potential for experience-dependent plasticity. From a metabolic point of view, it would be important to confirm in humans the finding of animal studies that changes of beta power reflect lactate consumption (Grønli et al., 2016). If true, this finding could be on one hand, the key to understand symptoms of neurological diseases in terms of energy regulation, from parkinsonian bradykinesia to fatigue (which is present not only in MS but also in several brain diseases) and to attention-related problems characteristic of neuropsychiatric disorders. On the other hand, it will also be particularly relevant for interventions geared to enhance the processes involved in LTP induction and maintenance that may deficient or abnormal in disorders characterized by neurodegeneration, inflammatory processes, maladaptive plasticity, and abnormal beta modulation. Topographical and connectivity studies about the temporal profile of beta ERD and ERS across sensorimotor (Meziane et al., 2015) and frontal areas during performance and practice would provide the bases for designing specific spatial and temporal neuromodulation interventions to improve performance. In terms of mechanisms, a firm definition of the link between beta power and center-surround suppression during motor and cognitive performance would be of paramount importance to understand attention-related problems in health and disease (Schmidt et al., 2019), and thus to address symptoms with novel approaches.

These are only a small sample of the many facets that need to be uncovered by research on movement-related beta ERD and ERS. Reaching these goals, an effort that needs a comprehensive multimodal translational approach, is essential to define targeted therapeutical interventions for the care of many motor and non-motor features of neuropsychiatric diseases.

## Author contributions

ET, MFG, and AQ contributed to the conception of the study. MFG, FF, JP, SG, CC, TA, SS, and SAS wrote the manuscript. MFG, JP, FF, DM, ET, and AQ revised the manuscript. All authors read and approved the submitted version.
